# Interferon alpha antagonizes the anti-hepatoma activity of the oncolytic virus M1 by stimulating anti-viral immunity

**DOI:** 10.18632/oncotarget.15788

**Published:** 2017-03-01

**Authors:** Liu Ying, Hu Cheng, Xu Wen Xiong, Lin Yuan, Zhang Hai Peng, Zhong Wen Wen, Liang Jian Ka, Xiao Xiao, Cai Jing, Tan Ya Qian, Gao Zhi Liang, Yan Guang Mei, Zhu Wen Bo, Peng Liang

**Affiliations:** ^1^ Department of Infectious Diseases, Third Affiliated Hospital of Sun Yat-Sen University, Guangzhou, Guangdong, China; ^2^ Guangdong Key Laboratory of Liver Disease Research, The Third Affiliated Hospital of Sun Yat-Sen University, Guangzhou, China; ^3^ Department of Urology Diseases, Third Affiliated Hospital of Sun Yat-Sen University, Guangzhou, Guangdong, China; ^4^ Department of Pharmacology, Sun Yat-Sen University, Guangzhou, China; ^5^ Department of Urology Diseases, The Sixth Affiliated Hospital of Sun Yat-Sen University, Guangzhou, Guangdong, China; ^6^ Department of Pharmacy, Third Affiliated Hospital of Sun Yat-Sen University, Guangzhou, Guangdong, China; ^7^ Collaborative Innovation Center for Cancer Medicine, Guangzhou, China

**Keywords:** hepatocellular carcinoma, oncolytic virus M1 virus, interferon, interferon-stimulated genes, ZAP

## Abstract

Alpha virus M1 is an oncolytic virus that targets zinc-finger antiviral protein (ZAP)-defective cancer cells, and may be useful for treatment of hepatocellular carcinoma (HCC). Most of HCC patients have hepatitis and need long-term antiviral medication. Thus, it is necessary to clarify whether anti-virus medicines influence oncolytic effect of M1. We examined the effect of drugs used to treat hepatitis B/C on M1-mediated oncolysis *in vitro* and *in vivo*. Interferon (IFN)-α induces expression of antiviral IFN-stimulated genes (ISGs) in HCC cells with moderate sensitivity to M1 virus. This leads to reduced replication of M1, and blocking of M1-mediated apoptosis. The antagonistic effect of IFN-α is positively related with the expressive level of ISGs. We also examined a population of 147 HCC patients. A total of 107 patients (73%) had low ZAP expression in liver tissues relative to adjacent tissues. Among these 107 patients, 77% were positive for hepatitis B and 2% were positive for hepatitis C. A combination of M1 virus and IFN should be avoided in those patients with HBV or HCV infection, of who ZAP expression is low but ISGs expression is moderate. In conclusion, this study provides a basis for anti-viral regimens for HCC patients with hepatitis B or C who are given oncolytic virus M1.

## INTRODUCTION

Hepatocellular carcinoma (HCC) is the sixth most common cancer and the second leading cause of cancer deaths worldwide [1–3].HCC accounts for 6% of the global incidence of cancer, and 9% of global cancer mortality. The incidence and mortality rates of HCC have increased over time due to the high mutation rates of these tumors, their ability to escape immune responses, their weak antigenicity, and the abundant blood supply to the liver [4].

The current approaches for treatment of HCC—surgery, local ablation, transcatheter arterial chemoembolization (TACE), chemotherapy, immunotherapy, and molecular targeted therapy are still unsatisfactory [5–7]. Furthermore, there is only limited support for modern interventions for treatment of HCC, in contrast to other common cancers. Therefore, there is an urgent need to identify new strategies to improve the survival of HCC patients.

A natural alphavirus M1, in the Togaviridae, was first isolated from Hainan Province, China in the 1960s [8, 9]. We previously reported that M1 selectively targets tumors deficient in the zinc-finger antiviral protein (ZAP), by causing prolonged and severe stress to the endoplasmic reticulum (ER) and cell apoptosis [10]. A safety evaluation in cynomolgus macaques [11] reported that 18 intravenous injections with M1 led to no toxicity based on clinical, biochemical, immunological, medical imaging, and other pathological analyses. This suggest that intravenous administration of oncolytic virus M1 may be safe for cancer patients. Furthermore, translational research that aims to use M1 in clinical practice are in progress. Importantly, we previously observed that M1 could kill HCC cells efficiently and selectively *in vitro*, *in vivo*, and *ex vivo*, and that it typically reduced the ZAP level in HCC tissues [10]. These results suggest that M1 has potential for use in HCC therapy.

Previous research reported that more than 50% of HCC patients worldwide have hepatitis virus infections, mostly hepatitis B virus (HBV) in China, South Korea, and Taiwan, and mostly HCV in North America, Europe, and Japan [12]. HBV and HCV infection is thus internationally recognized as a major cause of HCC, and also contributes to the recurrence and metastasis in HCC [13–15]. There is also evidence that surgical resection, TACE, radiation therapy, chemotherapy, and ablation therapy can activate HBV/HCV replication [15]. Therefore, international and domestic guidelines for HCC treatment support the need for anti-hepatitis treatments to reduce HBV/HCV viral loads and improve the prognosis of patients with virus-related HCC. A possible future therapy may be the simultaneous administration of anti-HBV/HVC agents and the M1 oncolytic virus for patients with HBV/HCV related HCC. Thus, it is essential to investigate whether anti-hepatitis drugs affect the oncolytic activity of M1 targeted therapy in HCC.

In this study, we examined the effect of multiple anti-HBV/HVC drugs on the oncolytic activity of M1 virus. These drugs included 3 classifications: ① first-line drugs used to treat clinical HBV infection, such as oral nucleoside analogues Entecavir [ETV], Lamivudine [LAM], Adefovir [ADV], Telbivudine [LDT], and Tenofovir [TDF]; ② new therapeutic drugs recommended for treatment of HCV infection in Europe and the United States, such as Daclatasvir [DCV], Telaprevir [TEL] and Sofosbuvir [SOF]); ③ broad antiviral drugs such as interferon alpha (IFN-α) and ribavirin (RBV).

## RESULTS

### Drugs against HBV and HCV do not weaken the oncolytic effect of M1 virus in HCC cells

We initially classified different HCC cell lines (high, mid, or low) according to their sensitivity to M1. For high-sensitive cells (Hep-3B), M1 inhibited cell viability by more than 75%. For mid-sensitive cells (Huh-7, Huh-6, sun-387, sun-449, sk-hep-1, sun-182, and Li-7), M1 inhibited cell viability by 25–75%. For low-sensitive cells (PLC, Hep-G2 and Bel-7420), M1 inhibited cell viability by less than 25%. Thus, for subsequent experiments we used Hep-3B, Huh-7, and PLC cells as high-, mid- and low-sensitive groups, respectively.

**Figure 1:**
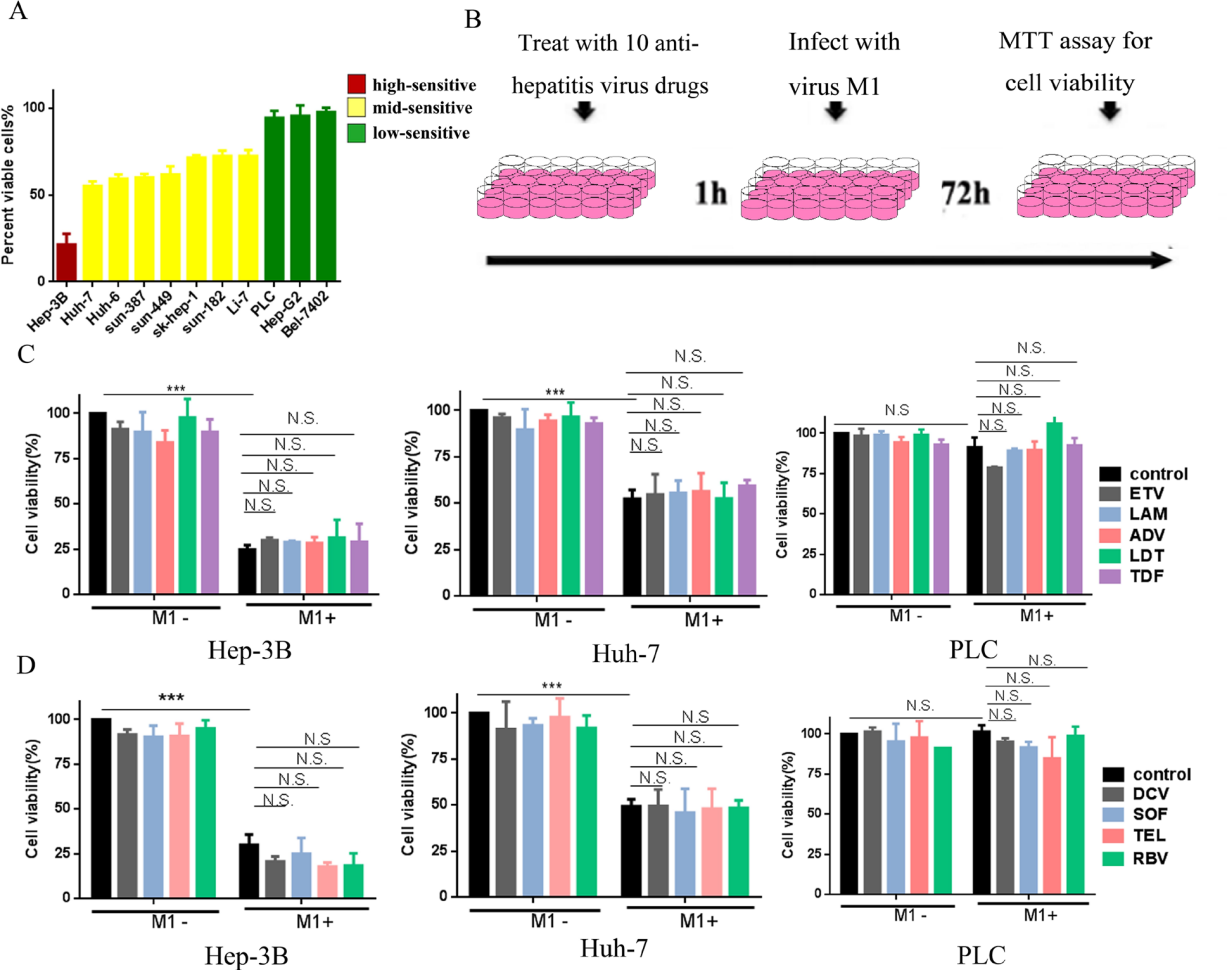
Common anti-virus chemicals for hepatitis combined with M1 virus don't antagonize the oncolytic effect in HCC cells (**A**) Cells were infected with (MOI = 10) M1 and cell viabilities were determined 48 hours post infection. For each cell line, the percentages of cell viabilities are color-coded by quartile. (**B**) Schematic representation of cell experimental process. (**C**, **D**) The indicated liver cancer cell lines—Hep-3B, Huh-7 and PLC were treated with or without 5 types anti-hepatitis B virus drugs (C), anti-hepatitis C virus drugs (D) and Ribavirin (D) with the concentration of 100 μM, and M1 virus (MOI = 10) for 72 hours. Following 72 hours, cell viabilities were determined by MTT assay (mean ± SD). N.S. Not significant.

We investigated whether anti-hepatitis drugs influence the oncolytic activity of M1 by treating Hep-3B, Huh-7, and PLC cells with 9 drugs commonly used against HBV (ETV, LAM, ADV, LDT, and TDF) and HCV (DCV, TEL, SOF, and RBV). Following the pretreatment with each drug for 1 h, cells were infected with M1 virus for another 72 h in combination with the drug (Figure 1B). Figures 1C and 1D show that M1 virus (10 MOI) induced loss of viability in Hep-3B and Huh-7 cells, but had little effect in PLC cells. Moreover, administration of any single anti-hepatitis drug in the range of 0.01 to 100 μM had no effect on cell viability in all tested cells (Figure 1C, 1D and Supplementary Figure 1). The highest concentration of these anti-hepatitis drugs is 100 uM, which is at least 10 times higher than IC50/EC50 of these drugs [16–24]. When cells were treated with a combination of M1 virus and drug, there were no significant differences between M1 alone and the combination (Figure 1C, 1D and Supplementary Figure 1). These results suggest that drugs commonly used against HBV and HCV do not antagonize the oncolytic activity of M1 virus in high-, mid- and low-sensitive HCC cells.

### IFN-α, the only biological agent against HBV and HCV, completely abrogates cell killing by M1 virus in mid-sensitive HCC cells but not in high-sensitive ones

**Figure 2:**
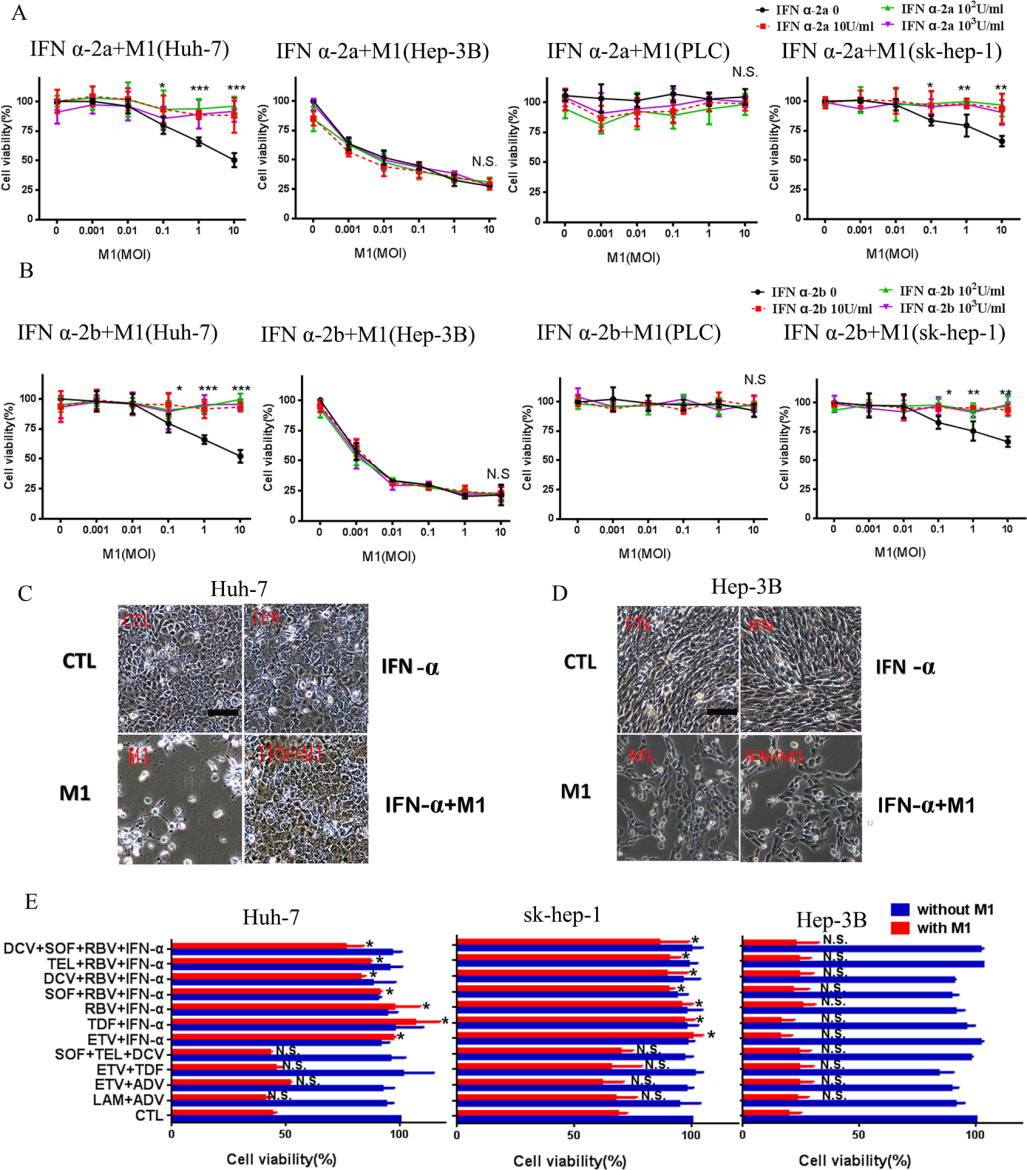
IFN-α inhibits the oncolytic effect of M1 in mid-sensitive HCC cells (**A**, **B**) INF α-2a (A) and INF α-2b (B) cancels the oncolytic effect of M1 in mid-sensitive hepatoma cells in Huh-7 and sk-hep-1, but don't antagonize in Hep-3B and PLC. Cells were treated with INF α-2a and INF α-2b with the concentration of 10U/ml, 10^2^U/ml and 10^3^U/ml. Cell viabilities were determined by MTT assay (mean ± SD). (**C**, **D**) Huh-7 (C )and Hep-3B (D) Cells were pretreated or non-pretreated with 10^3^IU/ml INF α-2a for 1 hour, then, cells were infected with 10 moi M1 virus for 72 hours (Scale bars: 100 μm.). (**E**) Huh-7, sk-hep-1and Hep-3B cells treated with combined anti-hepatitis therapy for 1 hour, and 10moi M1 virus for 72h. Following 72 hours, cell viabilities were determined by MTT assay (mean ± SD). CTL, control; N.S. Not significant. *P < 0.05; **P < 0.01, ***P < 0.001.

IFN-α, a type of Interferon-I, functions as a pivotal stimulator of anti-virus immunity, and is commonly used to treat patients with HBV and HCV infections. IFN α-2a and IFN α-2b are two common types of IFN-α, so we tested the effect of each on the oncolytic activity of M1. Figure 2A and 2B shows that IFN α-2a and IFN α-2b each completely abrogated the effect of M1 virus in Huh-7 cells, but not in Hep-3B and PLC cells. To verify this effect, we performed the same experiment in another mid-sensitive cell line sk-hep-1.

IFN-α is often used in conjunction with other anti-viral drugs, so we also examined the possible effects of multi-drug combinations, with or without IFN-α, on the oncolytic activity of M1. The 12 commonly used combination treatments were: LAM+ADV, ETV+ADV, ETV+TDF, SOF+TEL+DCV, ETV+IFN, TDF+IFN, RBV+IFN, SOF+RBV+IFN, DCV+RBV+IFN, TEL+ RBV+IFN and DCV+TEL+RBV+IFN [14, 15]. The data show that all regimens with IFN-α abrogated the oncolytic effect of M1 in Huh-7 and sk-hep-1 cells, but not in Hep-3B cells (Figure 2E). These data indicate that IFN-α strongly inhibits the oncolytic activity of M1 virus.

### IFN-α inhibits M1 virus by induction of specific genes in mid-sensitive HCC cells

**Figure 3:**
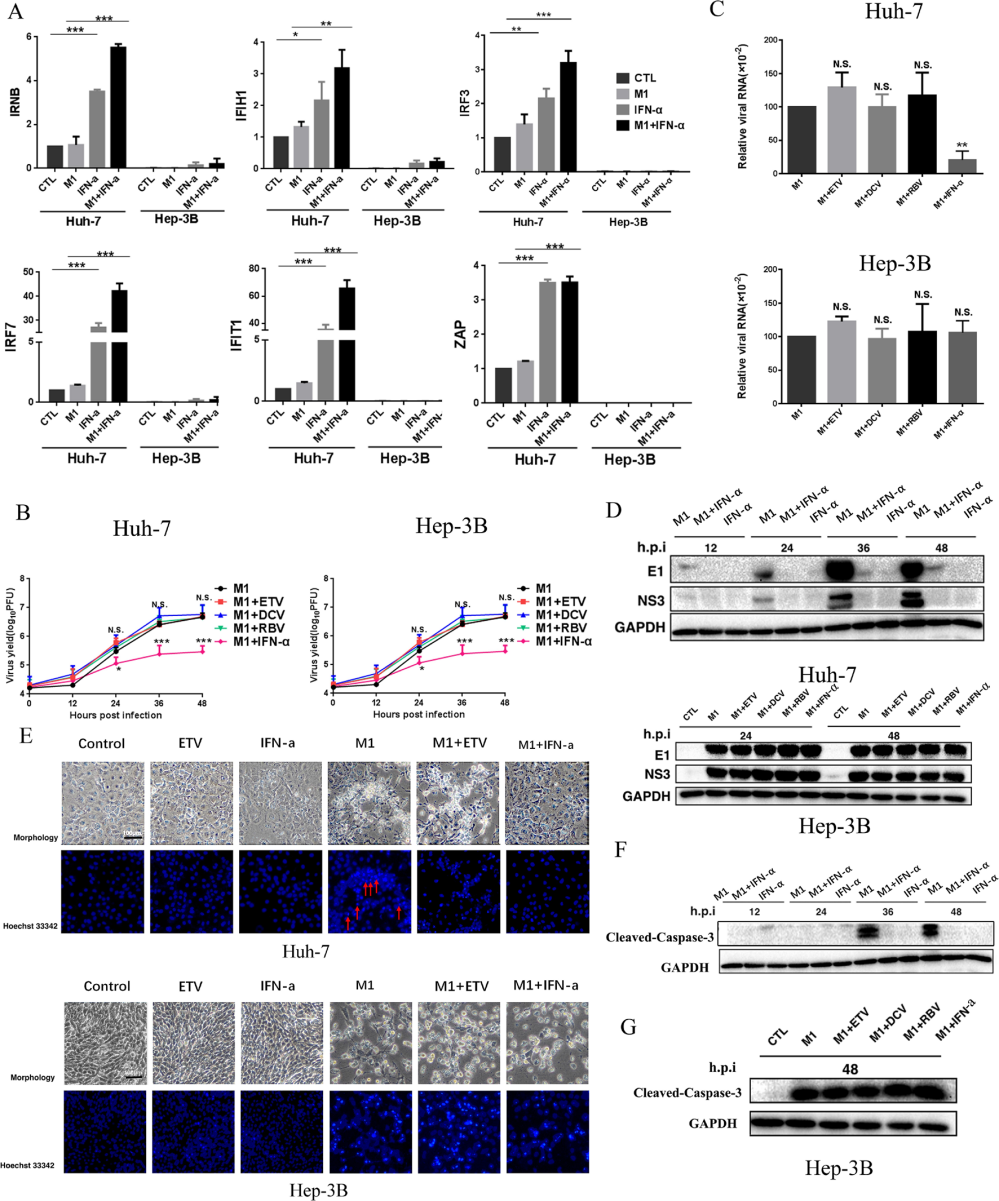
IFN-α activates M1 virus-induced antiviral factor expression and depresses the replication of M1 virus thus leading to the inhibition of cell apoptosis in mid-sensitive HCC cells (**A**) Huh-7 and Hep-3B cells were infected with M1 virus (10 PFU/cell) in the presence or absence of IFN a-2a (10^3^IU/ml), and IFNB, IFIH1, IRF3, IRF7, IFIT1 and ZAP mRNA levels were quantified by reverse transcription-polymerase chain reaction at 12 hours after M1 infection (mean ± SD). Fold-expression of genes was normalized to β-actin. (**B**) Viral titer determination in Huh-7 and Hep-3B lines (mean ± SD). (**C**) Huh-7 and Hep-3B cells were treated with M1 (MOI = 0.01 pfu per cell) for 24 h. The levels of viral genomic RNA and endogenous control β-actin were analyzed by qRTPCR. (mean ± SD) *P < 0.05, **P < 0.01, ***P< 0.001. (**D**) Western blots showing the expression of viral proteins E1 and NS3 24 hours post infection. GAPDH, glyceraldehyde-3-phosphate dehydrogenase. (**E**) INF α-2a would inhibit apoptosis of cancer cells which M1 causes in Huh-7 cell line, but not in Hep-3B cell line, Huh-7 and Hep-3B cells were treated with 1moi M1 (0.01moi M1 for Hep-3B), ETV, IFN-α or M1 plus anti-hepatitis virus drugs for 72 hours, then cells were stained by hochst33342 and photographed. (**F**) Expression of cleaved-caspase-3. Huh-7 Cells were treated with 1moi M1, IFN-α or M1/IFN-α combination for 12, 24, 36 and 48 hours, western blotting was performed to detect the candidate proteins. (**G**) Expression of Cleaved-Caspase-3. Hep-3B Cells were treated with 0.01moi M1 or M1/ETV, DCV, RBV, IFN-α combination for 48 hours, western blotting was performed to detect the candidate proteins. GAPDH, glyceraldehyde-3-phosphate dehydrogenase.

IFN-α, a classic antiviral drug, exerts its antiviral and immunomodulatory function by altering the expression of interferon-stimulated genes (ISGs) [16].Therefore, we determined whether the ISGs induced by IFN-α are responsible for its antagonistic effect against M1.Thus, we measured the RNA levels of 6 representative ISGs (IFNB, IFIH1, IRF3, IRF7, IFIT1, and ZAP) following exposure to M1, IFN-α, or M1 + IFN-α. In the mid-sensitive Huh-7cells, M1 slightly increased together, the M1 + IFN-α group displayed the strongest inducible activity. In high-sensitive Hep-3B cells, these ISGs were undetectable in controls and IFN-α only led to a slight increase in expression (Figure 3A). These results suggest a relationship between the deficient expression of ISGs of HCC cells and their sensitivity to M1.

Consistent with the data above, IFN-α but no other anti-viral drugs decreased M1 viral titers(Figure 3B), the levels of M1 viral genomic RNA (Figure 3C) and the levels of M1 structural and non-structural proteins (E1 and NS3) (Figure 3D) only in Huh-7 cells. These results indicate that IFN-α induced antiviral immunity, thus inhibiting replication of M1 virus.

### IFN-α represses cell apoptosis triggered by M1 virus

We also examined the effect of IFN-α on repression of M1-mediated apoptosis. Thus, we added M1 alone and in combination with ETV to Huh-7 and Hep-3B cells. The cells exhibited typical features of apoptosis, with a dramatic loss of cells and condensation of nuclei. However, addition of IFN-α abrogated all these effects in Huh-7 cells, but not Hep-3B cells (Figure 3E). In agreement, the level of Cleaved-Caspase-3 increases with incubation time of M1, but IFN-α blocked this effect in Huh-7 cells (Figure 3F). In Hep-3B cells, neither ETV, DCV, RBV, nor IFN-α affected the M1 mediated activation of Cleaved-Caspase-3 (Figure 3G). These results are consistent with data showing that IFN-α inhibits M1 virus replication in mid-sensitive hepatoma cells.

### IFN-α attenuates M1 virus activity in hepatoma xenografts from mid-sensitive cells, but not sensitive cells

**Figure 4:**
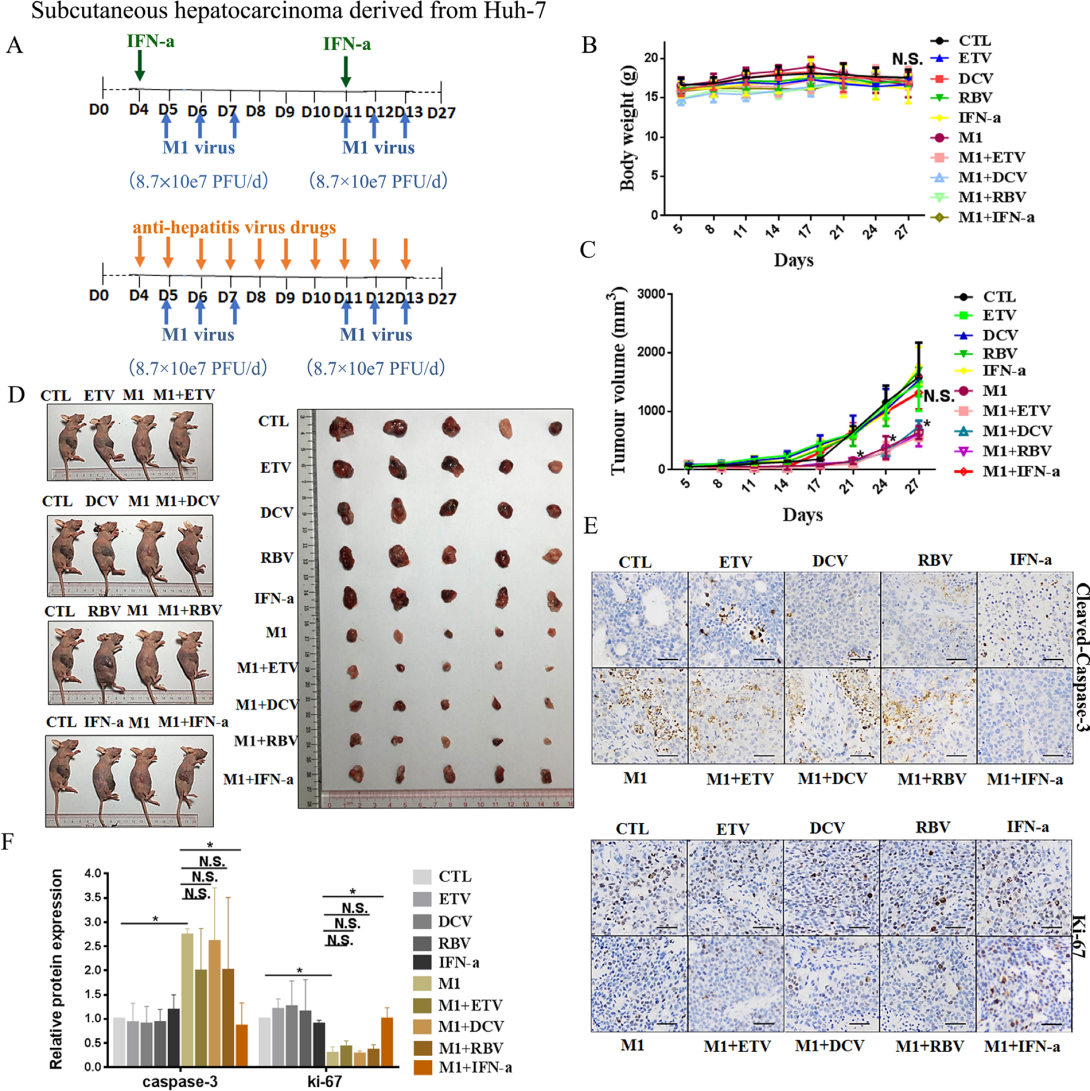
IFN-α attenuates anti-tumor activity of M1 virus invivo subcutaneous Huh-7tumors (**A**) Nude mice (NU/NU) bearing subcutaneous Huh-7 tumors were treated with vehicle ETV (75 μg/kg/day, i.p.), DAC (15 mg/kg/day, i.p.), RBV (15 mg/kg/day, i.p.) IFN-α (35μg/kg/week, s.c.), M1 virus (8.7 × 10^7^ PFU/day, i.v.), M1 virus and anti-hepatitis virus drugs. i.p.intraperitoneal injection, i.v., intravenously injection (tail vein), s.c. subcutaneous injection, PFU, plaque forming unit.(B and C)Body weight (**B**) and Tumor growth (**C**) of tumor-bearing mice. Data are shown in means ± SDs. N.S. Not significant. *P < 0.05, compared with the combination group. (**D**) At experimental endpoints, mice were anesthetized and sacrificed. Tumors weresubsequently dissected and photographed. (**E**)Intratumoral expression of Ki-67 and Cleaved-Caspase-3. (**F**) Immunohistochemistry was performed to analyze the expression of Ki-67 and Cleaved-Caspase-3. Relative protein expressions were quantified with Image-Pro Plus 6.0 (IPP 6.0) N.S., not significant. *P < 0.05.

**Figure 5:**
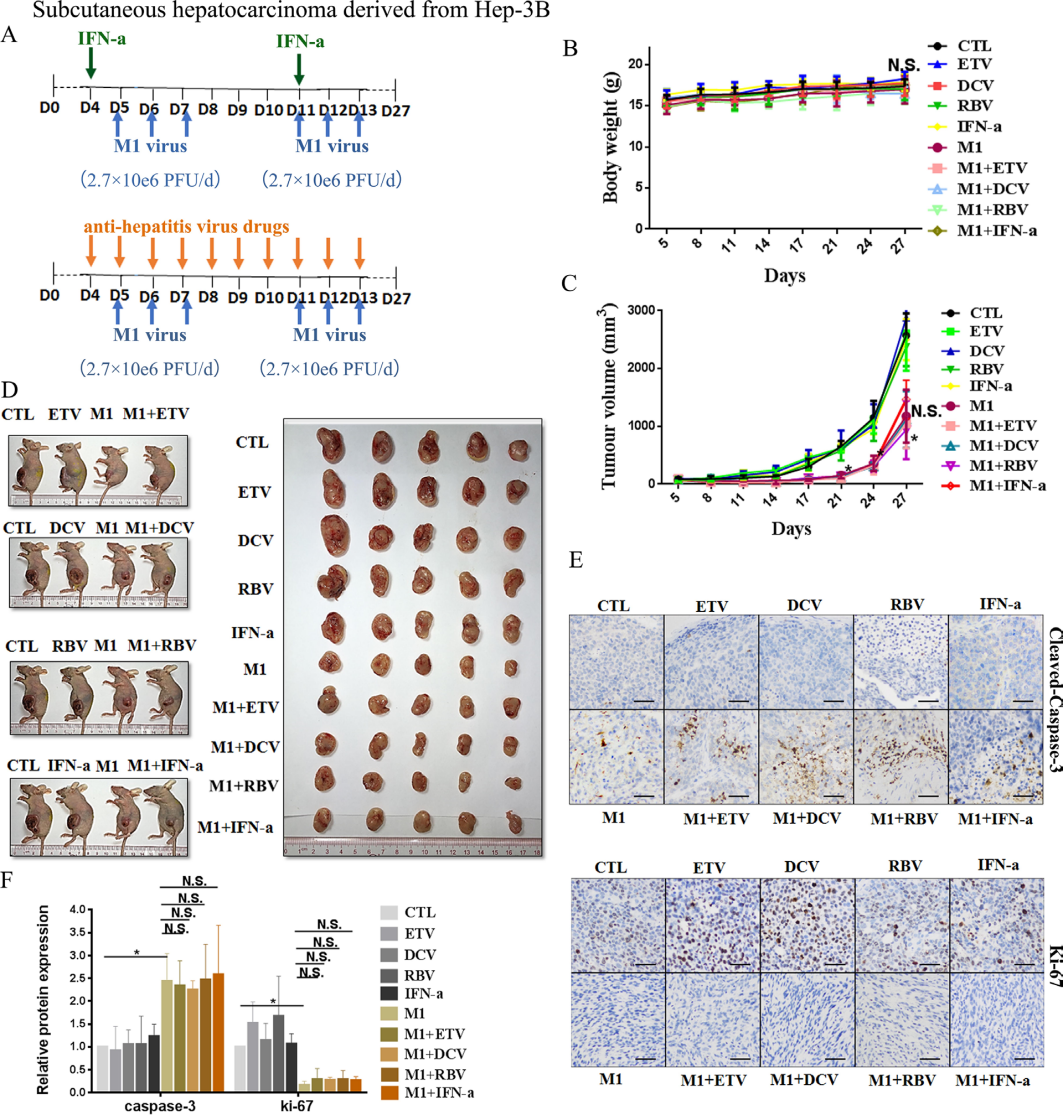
IFN-α attenuates anti-tumor activity of M1 virus *in vivo* subcutaneous Hep-3B tumors (**A**) Nude mice (NU/NU) bearing subcutaneous Hep-3B tumors were treated with vehicle, ETV (75 μg/kg/day, i.p.), DAC (15 mg/kg/day, i.p.), RBV (15 mg/kg/day, i.p.) IFN-α (35μg/kg/week, s.c.), M1 virus (2.7 × 10^6^ PFU/day, i.v.), M1 virus and anti-hepatitis virus drugs. i.p. intraperitoneal injection, i.v., intravenously injection (tail vein), s.c. subcutaneous injection, PFU, plaque forming unit. (**B** and **C**) Body weight (B) and Tumor growth (C) of tumor-bearing mice. Data are shown in means ± SDs. N.S. Not significant. *P < 0.05, compared with the combination group. (**D**) At experimental endpoints, mice were anesthetized and sacrificed. Tumors were subsequently dissected and photographed. (**E**) Intratumoral expression of Ki-67 and Cleaved-Caspase-3. (**F**) Immunohistochemistry was performed to analyze the expression of Ki-67 and Cleaved-Caspase-3. Relative protein expressions were quantified with Image-Pro Plus 6.0 (IPP 6.0) N.S., not significant. *P < 0.05.

We further evaluated the inhibitory effect of IFN-α on the oncolytic activity of M1 virus *in vivo* by establishing subcutaneous xenograft models derived from Huh-7 and Hep-3B cells in BALB/c-nu/nu mice. We divided the xenograft-bearing mice into 10 groups (5 mice per group) according to treatment (CTL, ETV, DCV, RBV, IFN-α, M1, M1+ETV, M1+DCV, M1+RBV, M1+IFN-α). After palpable tumors had formed, we randomized mice to receive injections of vehicle, ETV, DVC, RBV, or IFN-α, with or without M1 (Figures 4A and 5A). All mice were asymptomatic, did not lose body weight during the observation period (Figures 4B and 5B), and were sacrificed at 27 days after inoculation.

In agreement with the *in vitro* experiments, administration of M1 alone in Huh-7 derived xenografts at a higher dose (8.7 × 10^7^ PFU/day) significantly repressed the growth of Huh-7-derived xenografts, based on size of the tumor (Figure 4C), slowed tumor growth (Figure 4D), increased the level of cleaved Caspase 3, and decreased the level of Ki-67, a marker of cell proliferation (Figure 4E and 4F). We found no differences between the ETV/DCV/RBV plus M1 groups and the M1 alone group (Figure 4C–4F). However the IFN-α plus M1 group differed greatly from the M1 group, indicating that IFN-α also hampers the oncolytic activity of M1 *in vivo* (Figure 4C–4F).

Administration of M1 virus (2.7 × 10^6^ PFU/day) to Hep-3B derived xenografts remarkably slowed tumor growth (Figure 5C and 5D), but had no effect on overall body weight (Figure 5B). Treatment with M1 virus increased the number of cells with positive signals of cleaved Caspase 3, and decreased the number of cells with positive signals of Ki-67 (Figure 5E and 5F). Neither ETV, DCV, RBV, nor IFN-α altered the oncolytic efficacy of M1 virus in these cells (Figure 5B and 5F), in accordance of the *in vitro* data.

### HCC patients with HBV/HCV infection and negative expression of ZAP

**Figure 6:**
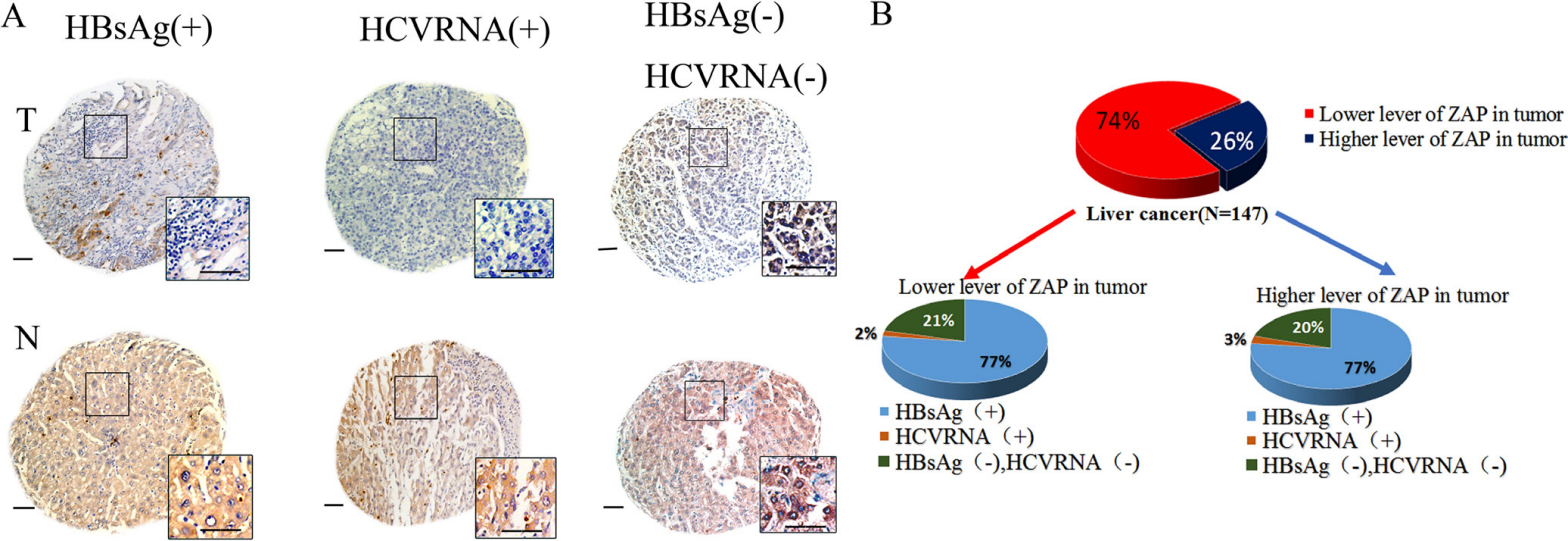
Clinical investigation on patients with negative ZAP and positive HBV/HCV (**A**) Representative cores of ZAP immunostaining in TMA. Higher magnification shown inthe box. (Scale bars: 50 μm.) N, nonneoplastic; T, tumor. (**B**) Statistical analysis of the expression of ZAP of tumor and serum HBsAg and HCVRNA of patients.

Our *in vitro* and *in vivo* results show that IFN-α blocks the anti-tumor activity of M1 virus in mid-sensitive HCC cells. This suggests possible harm from administration of an IFN-α + M1 combination regimen to HCC patients with hepatitis. We further investigated this topic by performing a molecular pathology study of 147 HCC patients. In particular, we determined ZAP expression and HBV/HCV infection status in 147 paraffin-embedded archived HCC tissues (Figure 6A). As we reported previously [8], a lower expression of ZAP implies that the patient is more likely to benefit from M1 virus therapy. The presence of HBV/HCV in tumor tissue means that the patient is likely to be given IFN-α therapy.

We performed ZAP immunohistochemistry assays in 8 tissue microarrays (TMAs) containing paired specimens (tumor and adjacent tissue) from all 147 patients. Our results show that ZAP had low expression in HCC tissues relative to adjacent tissues in 107 patients (73%). Among these 107 samples, 82 (77%) were positive for hepatitis B surface antigen (HBsAg), and 2 (2%) were positive for HCV RNA (Figure 6B). These 84 patients could possibly suffer harm if given a therapy consisting of IFN-α and M1 virus.

## DISCUSSION

Treatment guidelines for HCC instructs must consider benefits and harms, so patients with concomitant HBV/HCV infections are often given long-term antivirus therapy. The oncolytic virus M1 has great potential for use in HCC therapy, so it is essential to assess the effect of anti-viral drugs on the oncolytic activity of M1 before its clinical use. Our study shows that IFN-α suppresses the oncolytic effect of M1 virus in mid-sensitive HCC cells, but that nucleotide/nucleoside HBV analogues, and DAAs for HCV and RBV did not inhibit M1-induced oncolysis. Importantly, these results provide basic information regarding the use of suitable drug combinations to be used with the M1 oncolytic virus for treatment of HCC patients with HBV/HCV infections.

Clinicians commonly use IFN-α to treat patients with chronic HBV and HCV infections. Our study serves as a proof-of-concept that IFN-α suppresses the oncolytic effect of the M1 virus. Thus, for HCC patients with newly diagnosed HBV or HCV infections and considering M1 oncolytic therapy, IFN-α should be avoided to improve the efficiency of M1 virus. Moreover, for HCC patients who are taking IFN-α as an anti-hepatitis therapy, this drug should be altered into non-IFN antiviral agent when considering treatment with M1 virus. Our data showing an antagonism of IFN-α with M1 serves as a strong warning for the future development of M1 virus therapy. Previous research reported that IFN-α inhibited replication and infection of vesicular stomatitis virus (VSV) in murine and human brain cultures, and *in vivo* mouse models [25]. VSV also shows promise as an oncolytic virus for treatment of HCC [3]. Dold et al. found that an inhibitor of the interferon response overcame the partial resistance of human ovarian cancers to VSV oncolytic therapy [26]. Although there is no direct evidence that IFN-α inhibits the oncolytic activity of VSV, our research suggests this is a possibility.

IFN-α activates the expression many ISGs, leading to inflammation and host antiviral responses [27]. Previous studies showed that IFN can inhibit oncolytic herpes simplex virus (oHSV), as indicated by upregulation of ISGs in normally permissive cells, and a significant decrease of oHSV proliferation [28]. In agreement with these data on oHSV, we demonstrated that IFN-α activated ISGs (IFNB, IRF3, IRF7, IFIH1, and IFIT1) are responsible for the inhibition of M1 virus replication and blocking apoptosis. These results provide a reasonable interpretation for the antagonistic effect of IFN-α on M1 therapy.

It is worth noting that the antagonistic effect of IFN-α on M1 differs between mid-sensitive and high-sensitive HCC cells. Our *in vitro* and *in vivo* results show that IFN-α blocked the effect of M1 in mid-sensitive cells, but not in high-sensitive cells. Our previous studies indicated that low expression or deficiency of ZAP in cancercells accounts for the high-sensitive and oncolytic efficacy of M1 [10, 29]. Thus, we assumed that IFN-α may induce the expression of ZAP (an ISG), thus leading to inhibition of M1-induced oncolysis. Our additional experiments showed that although IFN-α stimulated the expression of ZAP mRNA and protein in mid-sensitive Huh7 cells, but not high-sensitive Hep3B cells. However, silencing of ZAP by siRNA in Huh7 cells did not affect the antagonistic effect of IFN-α on M1 (Supplementary Figure 2). These results indicate that stimulation of ZAP is not predominantly responsible for the effect of IFN-α on M1-induced oncolysis. Our results suggest that stimulation of multiple ISGs (IFNB, IRF3, IRF7, IFIH1, and IFIT1) are likely responsible for the anti-viral activity of IFN-α.

Intriguingly, we observed that high-sensitive cells have low or no detectable expression of all tested ISGs (IRF3, IRF7, IFIH1, IFIT1, IFNB, and ZAP), in parallel with their resistance to IFN-α. Loss of anti-viral responses through down-regulation of ISGs could explain why IFN-α did not impair the oncolytic activity of M1 in these high-sensitive cells. As a contrast, our PCR results indicate that ISGs are expressed in of mid-sensitive HCC cells, that IFN-α alone increases their expression, IFN-α+M1 further increases their expression. Our study also shows that the antagonistic effect of IFN-α on the oncolytic activity of M1 positively correlated with the level of ISGs. About 65–70% of tumor cell lines have interferon (IFN) response defects [2, 30]. The lack of an innate immune response makes it easier for viruses to enter cells and replicate.

Naturally occurring oncolytic viruses, such as human Respiratory Syncytial virus, Newcastle disease virus, and Malabar virus, have therapeutic potential because massive replication of these pathogens can ultimately kill tumor cells [1–3]Considering that gene products in the IFN-α-stimulated pathway are frequently defective in cancer, ISGs may be useful as biomarkers for individualized treatments. Thus, in the future, HCC patients with HBV/HCV infectious could be divided into ISGs+ and ISGs- groups, so that only the latter group is suitable for concurrent administration of IFN-α and M1.

Our research indicated that anti-HBV drugs (ETV, LAM, ADV, LDT, and TDF), DAAs for HCV (DCV, TEL and SOF), and the broad-spectrum anti-viral drug RBV did not inhibit the oncolytic effect of M1. These data imply that these drugs can be recommended even when M1 virus therapy is prescribed for HCC patients. As previously shown, these drugs achieve their effects by acting as competitive inhibitors of certain viral proteases, DNA polymerases, RNA polymerases, reverse transcriptase, or other targets. Thus, in theory, these agents should have strong antivirus effects. However, they are only virus inhibitors and cannot effectively eradicate HBV or HCV completely during the acute period of viral infection [31]. Our results show that these anti-virus drugs also had weak activity against M1.

We found that IFN-α antagonized the oncolytic effect of M1 virus by stimulating the expression of anti-viral genes in human hepatoma cells. By reviewing the medical history and histopathological chip results of 147 HCC patients, we found that more than 70% of these patients had low expression of ZAP, and 79% were positive for HBV or HCV infections. Thus, many HCC patients could initially be considered for simultaneous administration of anti-viral agents and M1 virus. However, our demonstration that IFN-α interferes with the oncolytic activity of M1 indicates that careful patient selection is needed before co-administration of IFN-α with M1 to HCC patients with HBV or HVC infections. Consequently, common anti-hepatitis regimen consisting of chemicals can be prescribed when patients are on the M1 virus therapy. Moreover, co-administration of IFN-α with M1 virus is not recommended for HCC patients with abundant expression of ISGs in their tumors.

## MATERIALS AND METHODS

### Cell lines, viruses, and reagents

Cell lines were maintained at 37°C with 5% CO2, in Dulbecco's modified Eagle's medium, supplemented with 10% (vol/vol) fetal bovine serum and 1% penicillin/streptomycin (Life Technologies). All cell lines were purchased from the American Type Culture Collection or the Shanghai Institute of Cell Biology.

The following reagents were used: ETV(Entecavir, 10 mmol/L, dissolved in dimethylsulfoxide [DMSO], S1252-10 mg, Selleck), LAM (Lamivudine, 10 mmol/L, dissolved in DMSO, S1706-10 mM in 1mL DMSO, Selleck), ADV(Adefovir 10 mmol/L, dissolved in DMSO, S1718-10mM in 1 mL DMSO, Selleck), LDT (Telbivudine 10 mmol/L, dissolved in DMSO, S1651-10 mg, Selleck), TDF (Tenofovir 10 mmol/L, dissolved in DMSO, S1401-10 mM in 1 mL DMSO, Selleck), DCV (Daclatasvir 10 mmol/L, dissolved in DMSO, S1482-5 mg, Selleck), TEL (Telaprevir 10 mmol/L, dissolved in DMSO, S1538-5 mg, Selleck), SOF (Sofosbuvir 10 mmol/L, dissolved in DMSO, S2794-5 mg, Selleck), RBV( Ribavirin 10 mmol/L, dissolved in DMSO, 10 mM in 1 mL DMSO, Selleck), IFN α-2a (Interferon alpha 2a, 10^7^ U/L, dissolved in 0.1% BSA, 300-02AA-100 μg, PeproTech), and IFN α-2b (Interferon alpha 2b, 10^7^ U/L, dissolved in 0.1% BSA, 300-02AB-100 μg, PeproTech).

### Cell viability assays

Cells were seeded in 24-well plates at 30,000 cells per well, and various drugs and M1 virus were added, as described in the figure legends. After 72 h, viability was determined by the 3-(4, 5-dimethylthiazol-2-yl)-2, 5-diphenyltetrazolium bromide (MTT) assay. In particular, MTT was added to the cells (1 mg/mL final concentration), and cells were allowed to grow at 37°C for another 3 h. Media was removed, and precipitates were dissolved in 500 μL DMSO. The optical absorbance was determined at 570 nm using a microplate reader (iMark; Bio-Rad).

### Quantitative reverse transcription-polymerase chain reaction

Total RNA was extracted using the TRIzol reagent (Life Technologies), and reverse transcribed to cDNA with oligo (dT). Gene expression was quantified using SuperReal PreMix SYBR Green (FP204-02, TIANGEN, Beijing, China) on an Applied Biosystems 7500 Fast Real-Time PCR system (Life Technologies). Expression of all genes were normalized to β-actin. The amplification primers (Thermo Fisher) are: IFIH1sense (TCACAAGTTGATGGTCCTCAAGT), IFIH1antisense (CTGATGAGTTATTCTCCATGCCC); IRF3 sense (AGA GGCTCGTGATGGTCAAG), IRF3 antisense (AGGTCC ACAGTATTCTCCAGG); IRF7 sense (CCCACGCTATAC CATCTACCT), IRF7 antisense (GATGTCGTCATAG AGGCTGTTG); IFIT1 sense (TTGATGACGATGAAA TGCCTGA), IFIT1 antisense (CAGGTCACCAGACTCC TCAC); IFNB sense (GCTTGGATTCCTACAAAGA AGCA), IFNB antisense (ATAGATGGTCAATGCGG CGTC); ZAP sense (TCACGAACTCTCTGGACTGAA), ZAP antisense (ACTTTTGCATATCTCGGGCATAA); M1 NS3sense (GGGGAGGGCTTTCTTTGTCA), M1 NS3antisenseCACCCTGTCTTGTCTTTGCTG); β-actin sense (GATCATTGCTCCTCCTGAGC), β-actin antisense (ACTCCTGCTTGCTGATCCAC).

### M1 Virus

M1 was grown in Vero cells (OPTISFM, 12309-019, Thermo Fisher, Waltham, MA). Virus titer was determined using the TCID50 assay for BHK-21 cells, and converted to plaque forming units (PFUs). The variant of M1 in this study was described previously [8].

### Antibodies and western blot analyses

Cells were lysed using M-PER Mammalian Protein Extraction Reagent (Thermo Scientific), and SDS gel electrophoresis was then performed. The primary antibodies were: M1 E1 (Beijing Protein Innovation, Beijing, China), NS3 (Beijing Protein Innovation, Beijing, China), ZAP (PA5-31650; Thermo Scientific), GAPDH (AP0060; Bioworld), β-actin (AP0063, Bioworld), and Cleaved-Caspase-3 (9664s, Cell Signaling Technology). Treatment with each primary antibody was followed by treatment with a HRP-conjugated secondary antibody. Membranes were visualized on a ChemiDoc XRS+ System (Bio-Rad), using the Immobilon Western Chemiluminescent HRP Substrate (Millipore).

### Hochst33342 staining

Cells were seeded in 96-well plates (4000 cells per well) and various drugs and M1 virus were added, as described in the figure legends. After 72 h, Hochst 33342 (Sigma-Aldrich, USA) was added (10% vol/vol final concentration), and cells were allowed to grow at 37°C for another 20 min. Then, the medium was removed, and cells were washed 3 times with sterile PBS. Cells were visualized by fluorescence microscopy (NIKON intersilight) and photographed.

### Animal models

All mouse studies were approved by the Animal Ethical and Welfare Committee of Sun Yat-sen University. Huh-7 cancer cells (5 × 10^6^ cells per mouse) and Hep3B cancer cells (5 × 10^6^ cells per mouse) were inoculated subcutaneously into the hind-flanks of 4-week old female BALB/c-nu/nu mice. Palpable tumors developed after 4 days (50 mm^3^), and mice were then randomly divided into 10 groups (5 mice per group): (i) intravenous vehicle alone, (ii)intravenous M1 (8.7 × 10^7^ pfu per dose M1 into Huh-7 subcutaneous xenograft mice, 2.7 × 10^6^ pfu per dose M1 into Hep-3B subcutaneous xenograft mice ), (iii) intraperitoneal ETV (75 μg/kg/day), (iv) intraperitoneal DCV(15 mg/kg/day), (v) intraperitoneal RBV(15mg/kg/d, ip), (vi) subcutaneous IFN-α (pegylated Interferon alpha 2a, 35μg/kg/week), (vii) intravenous M1 in combination with intraperitoneal ETV (viii) intravenous M1 in combination with intraperitoneal DCV, (ix) intravenous M1 in combination with intraperitoneal DCV, (x) intravenous M1 in combination with subcutaneous IFN-α. Every drugs in single injection are in a total volume of 200 μL. The dose of the combination groups are consistent with the dose of the single groups.

Tumor length and width were measured every other day, and the volume was calculated as (length × width^2^)/2. Mice were weighed every other day. All observers were blinded to group allocation.

### Immunohistochemistry assay

The expression of Cleaved-Caspase 3 (9664s, Cell Signaling Technology) and Ki-67(9449s, Cell Signaling Technology) in tumors were measured using specific antibodies. Briefly, tumor sections (4 μm) were dewaxed in xylene, hydrated in descending concentrations of ethanol, immersed in 0.3% H_2_O_2_-methanol for 30 min, washed with phosphate-buffered saline, and probed with monoclonal anti-Cleaved-Caspase 3 antibodies (1:100), Ki-67antibodies (1:100), or isotype control at 4°C overnight. After washing, the sections were incubated with biotinylated goat anti-rabbit or anti-mouse IgG at room temperature for 2 h. Immunostaining was visualized using the streptavidin/peroxidase complex and diaminobenzidine, and sections were then counterstained with hematoxylin. We quantified protein expression using Image-Pro Plus 6.0 software (MediaCybernetics).

### ZAP silencing and ectopic expression

For ZAP silencing, we used specific and non-targeting siRNAs that were synthesized by Ribobio (Guangzhou, China). Cells were replaced with 10% fetal bovine serum in DMEM (without penicillin/streptomycin). SiRNA transfection was performed using Lipofectamine RNAiMAX (13778-150, Thermo Fisher) with OPTIMEM (31985070, Thermo Fisher). Cells were transfected with 30 nM scrambled or ZAP siRNAs for 48 h, followed by exposure to M1 and various downstream effectors.

For ZAP overexpression, transient transfection was performed using FuGENE HD (Promega) according to the manufacturer's directions. Cells were transfected with pReceiver-M02 plasmids that expressed GFP (negative control) or ZAP (full length; GeneCopoeia) for 48 h, and then treated with M1.

### Clinical samples and clinical staging system

A total of 147 paraffin-embedded liver cancer samples were analyzed histopathologically and clinically at the Sun Yat-sen University Cancer Center (State Key Laboratory of Oncology in South China, Guangzhou, China). Serum HCV RNA viral load (IU/mL) was defined as negative when it was under the limit of detection (10^3^ IU/mL) based on a qPCR HCV-RNA test kit (Cobas V2.0, Roche). Serum hepatitis B surface antigen (HBsAg) was detected with an ELISA kit (KEHUANG Company, Shanghai).

### TMA

TMAs were provided by Dan Xie (State Key Laboratory of Oncology in South China, Sun Yat-sen University Cancer Center, Guangzhou, China). IHC staining was performed on 5-μm sections of the TMAs to assess cytoplasmic expression of ZAP (PA5-31650; Thermo Scientific). TMA slides were scanned using the Aperio slide scanner, and quantified using ImageScope software (Aperio).

IHC stains on tissues without necrosis were also scored by two independent pathologists as follows: score = proportion of positive stain (0, < 10%; 1, 10–25%; 2, 25–50%; 3, > 50%) × mean stain intensity (0–3).

### Statistical analysis

All statistical analyses were performed using SPSS 13.0 software (SPSS, IBM, Armonk, NY). Most comparisons employed Student's *t*-test or a one-way analysis of variance, followed by Dunnett's multiple post hoc test. Tumor volumes were analyzed by a repeated measures one-way analysis of variance. Unless otherwise indicated, all error bars indicate SD. A *P* value below 0.05 was considered significant.

### Study approval

All animal studies were approved by the Sun Yat-sen University Institutional Animal Care and Use Committee. Use of primary cancer tissue specimens was approved by an ethics review committee at Sun Yat-sen University.

## SUPPLEMENTARY MATERIALS FIGURES


