# Supplementary material for: Interferon alpha antagonizes the anti-hepatoma activity of the oncolytic virus M1 by stimulating anti-viral immunity
Source: Oncotarget. 2017 Mar 1;8(15):24694–705. doi: 10.18632/oncotarget.15788 (PMC5421880; doi:10.18632/oncotarget.15788)
Supplement: Supplementary file 1 [file oncotarget-08-24694-s001.pdf]

Interferon alpha antagonizes the anti-hepatoma activity of the oncolytic virus M1 by stimulating anti-viral immunity

Supplementary Materials

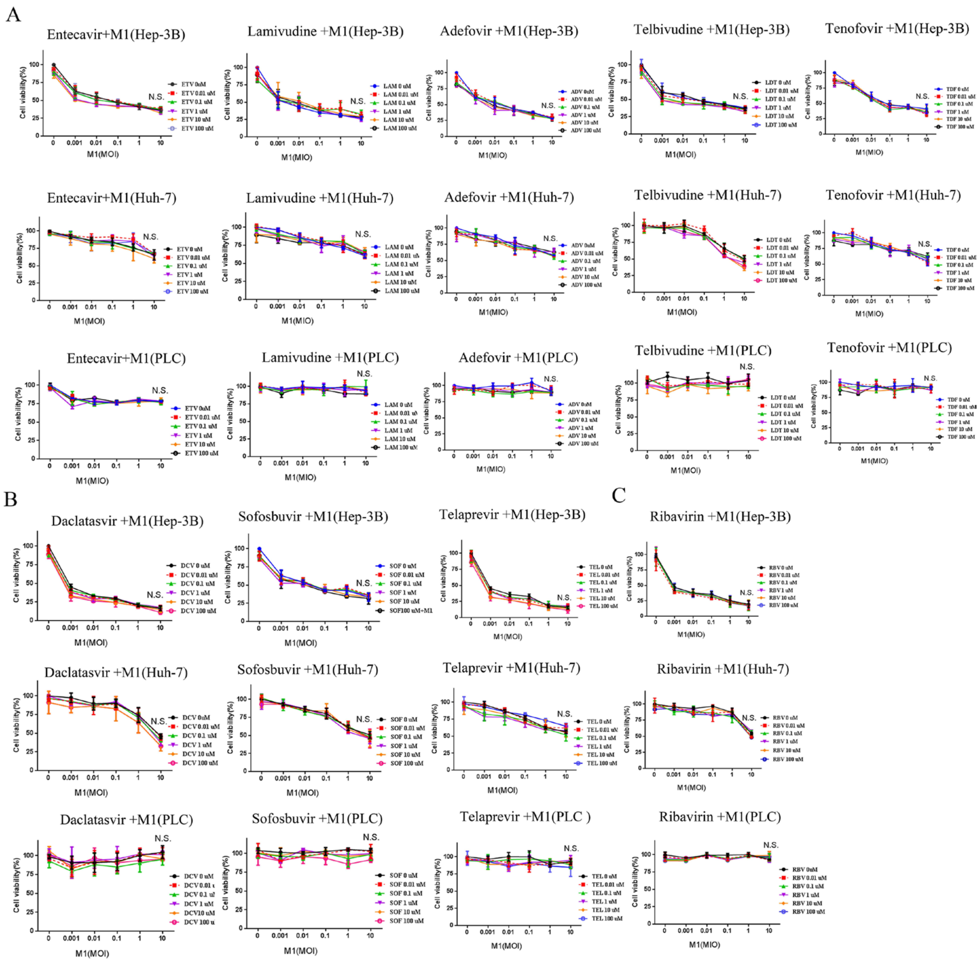

Supplementary Figure 1: The indicated liver cancer cell lines—Hep-3B, Huh-7 and PLC were treated with or without 5 types anti-hepatitis B virus drugs (**A**), anti-hepatitis C virus drugs (**B**) and Ribavirin (**C**) with the concentration of 0.01 μM, 0.1 μM, 1 μM, 10 μM, 100 μM and M1 virus (MOI = 0.001, 0.01, 0.1, 1, 10) for 72 hours. Following 72 hours, cell viabilities were determined by MTT assay (mean ± SD). N.S. Not significant.

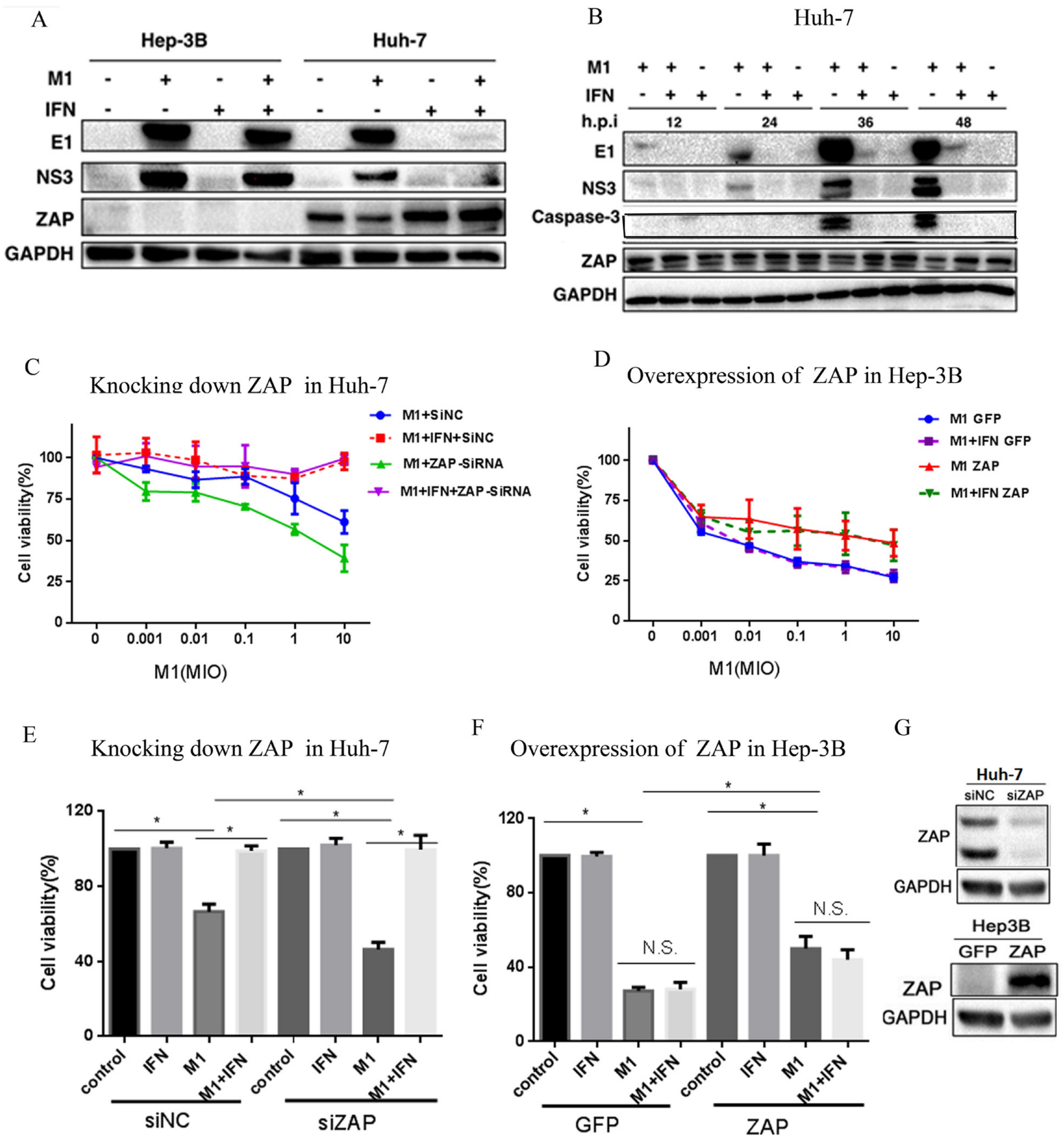

Supplementary Figure 2: (**A**) Hep-3B and Huh-7 cells were treated with vehicle, 0.01 moi M1, IFN-α or M1/IFN-α combination for 48 hours, western blotting was performed to detect the candidate proteins. (**B**) Huh-7 cells were treated with 0.01moi M1, M1/IFN-α combination or IFN-α for 12, 24, 36 and 48 hours, western blotting was performed to detect the candidate proteins. (**C**, **E**) Knockdown of ZAP did not affect the trend of inhibition of M1-induced oncolytic change. (**D**, **F**) Overexpression of ZAP in Hep-3B cells induce the trend of inhibition of M1-induced oncolysis change compared with negative control (GFP). (**G**) Huh-7 cells were transfected with small interfering RNAs (siRNAs) against ZAP. Hep-3B cells were transfected with plasmids expressing GFP (negative control) or ZAP for 48 h. ZAP expression levels were determined. GAPDH, glyceraldehyde-3-phosphate dehydrogenase. NC, negative control (scrambled siRNA). N.S., not significant. *$P < 0.05$.
